# Limiting resource and leaf functional traits jointly determine distribution patterns of leaf intrinsic water use efficiency along aridity gradients

**DOI:** 10.3389/fpls.2022.909603

**Published:** 2022-07-29

**Authors:** Jing Wang, Xuefa Wen

**Affiliations:** ^1^Key Laboratory of Ecosystem Network Observation and Modeling, Institute of Geographic Sciences and Natural Resources Research, Chinese Academy of Sciences, Beijing, China; ^2^Collaborative Innovation Center on Forecast and Evaluation of Meteorological Disasters (CIC-FEMD), Nanjing University of Information Science & Technology, Nanjing, China; ^3^College of Resources and Environment, University of Chinese Academy of Sciences, Beijing, China; ^4^Beijing Yanshan Earth Critical Zone National Research Station, University of Chinese Academy of Sciences, Beijing, China

**Keywords:** intrinsic water use efficiency, carbon stable isotope, oxygen stable isotope, environment filter, leaf economic spectrum

## Abstract

Intrinsic water use efficiency (iWUE) is a critical eco-physiological function allowing plants to adapt to water- and nutrient-limited habitats in arid and semi-arid regions. However, the distribution of iWUE in coexisting species along aridity gradients and its controlling factors are unknown. We established two transects along an aridity gradient in the grasslands of Losses Plateau (LP) and Inner Mongolia Plateau (MP) to elucidate the patterns and underlying mechanisms of iWUE distribution in coexisting species along aridity gradient. We determined leaf carbon (δ^13^C) and oxygen (δ^18^O) stable isotopes, functional traits related to carbon fixation, and limiting resources. Bulk leaf δ^13^C and δ^18^O were used as proxies for time-integrated iWUE and stomatal conductance (gs) during the growing season. Our results showed that variability in iWUE within transect was primarily controlled by species, sampling sites and an interactive effect between species and sampling sites. Mean values of iWUE (iWUE_Mean_) increased and coefficient of variation (CV) in iWUE (iWUE_CV_) decreased with an increase in aridity, demonstrating that increases in aridity lead to conservative and convergent water use strategies. Patterns of iWUE_Mean_ and iWUE_CV_ were controlled primarily by the ratio of soil organic carbon to total nitrogen in LP and soil moisture in MP. This revealed that the most limited resource drove the distribution patterns of iWUE along aridity gradients. Interspecific variation in iWUE within transect was positively correlated with Δ^18^O, indicating that interspecific variation in iWUE was primarily regulated by gs. Furthermore, relationship between iWUE and multi-dimensional functional trait spectrum indicated that species evolved species-specific strategies to adapt to a harsh habitat by partitioning limiting resources. Overall, these findings highlighted the interactive effects of limiting resources and leaf functional traits on plant adaptation strategies for iWUE, and emphasized the importance of considering biological processes in dissecting the underlying mechanisms of plant adaptation strategies at large regional scales.

## Introduction

Arid and semi-arid regions constitute ~41% of the global land area (Reynolds et al., 2007), and are predicted to expand in the future (Yao et al., 2020). Limited water and nutrient resources in these regions severely restrict plant growth, survival, and reproduction (Martin-StPaul et al., 2017). To avoid hydraulic failure and carbon starvation (Choat et al., 2018), plants open or close the stomata to balance carbon uptake and water losses (Galmes et al., 2007). Consequently, intrinsic water use efficiency (iWUE), the ratio of carbon gain in photosynthetic rate (A) to stomatal conductance (gs), is a critical eco-physiological strategy for plant water–carbon regulation in the process of adaptation to water- and nutrient-limited habitats (Moreno-Gutierrez et al., 2012; Querejeta et al., 2018; Wang et al., 2018).

Diverse trade-offs between carbon gain and water loss (iWUE) facilitate species coexistence in habitats with harsh and unpredictable environmental conditions (Moreno-Gutierrez et al., 2012; Bermúdez and Retuerto, 2014; Wang et al., 2018). In generally, plants with a conservative water use strategy (high iWUE) are better able to adapt to water-limited habitats than those with low iWUE (Flexas et al., 2016; Aparecido et al., 2020). However, opportunistic water-use strategy (low iWUE) is advantageous for nutrient acquisition (Querejeta et al., 2018). Along environmental gradients, limiting resources act as environmental filters, shape the expression of plant eco-physiological functions (Bahar et al., 2016), determine trait distribution of each species in the community, and theoretically drive species toward an “optimum” for a set of environmental conditions (Mitchell et al., 2018). However, understanding of iWUE distribution in coexisting species in response to aridity gradients, and its controlling factors remain incomplete.

Given that iWUE is regulated by a rapid response of A and gs to environmental variables (Prentice et al., 2014; Basu et al., 2021), time-integrated iWUE can reflect adaptability of plants to their environment, especially in areas with large environmental fluctuations (Maxwell et al., 2018; Guerrieri et al., 2019). Leaf carbon isotope discrimination during photosynthesis (Δ^13^C) is linearly related to the ratio of the partial pressures of intercellular (Ci) to ambient (Ca) CO_2_ (Ci/Ca), and is influenced by both A and gs over a period when the leaf is produced (Farquhar et al., 1989; Maxwell et al., 2018; Prieto et al., 2018). Time integrated iWUE can be estimated independently from the carbon isotope ratios (δ^13^C) of leaf tissue, allowing for more extensive studies of plant water use strategies at broader spatial scales than traditional leaf gas exchange measurements (Prentice et al., 2014).

According to the least-cost theory (Wright et al., 2003), water and nitorgen (N) are expected to be the most important environmental filters shaping the distribution of iWUE in each species in communities along aridity gradients. Previous studies conducted in arid and semi-arid areas focused primarily on spatial patterns of iWUE in response to water variables. As expected, leaf iWUE in drylands increases with decreasing precipitation (Zheng and Shangguan, 2007; Liu et al., 2013; Wang et al., 2016) or soil moisture (SM) (Ale et al., 2018) to reduce water cost. Alternatively, iWUE may decrease to reduce N cost when low soil N increases the cost of soil N uptake (Wright et al., 2003). In general, soil N supply (i.e., mineralization and nitrification rates) exhibits a decreasing trend with increasing water stress (Feyissa et al., 2021). However, it is not clear whether water or N-limitation dominates the distribution of iWUE in co-occuring species in communities along aridity gradients. In addition, evidence is accumulating in support of diverse water use strategies in resource-limited ecosystems (Moreno-Gutierrez et al., 2012; Bermúdez and Retuerto, 2014; Wang et al., 2018). However, it has not been established how this diversity varies with the strength of environmental filtering.

Large interspecific differences in iWUE along environmental gradients may be driven by variability in leaf functional traits within species (Maxwell et al., 2018; Rumman et al., 2018; Tang et al., 2021), because leaf functional traits can determine species persistence against strong environmental filters along a resource-use strategies gradient (Maracahipes et al., 2018; Guo et al., 2021; Li et al., 2022). Theoretically, many leaf functional traits correlate with variability in iWUE, including leaf N per unit area (Prentice et al., 2014) and specific leaf area (SLA) (Maxwell et al., 2018; Prieto et al., 2018). However, these are likely secondary to the primary effects of A and gs (Roden and Farquhar, 2012). In general, the first response of plants to dryness stress is to reduce gs, and photosynthetic capacity begins to decrease with the increase in dryness stress (Utkhao and Yingjajaval, 2015). However, it is challenging to determine whether variability in time-integrated iWUE derived from Δ^13^C is the result of changes in A, or gs, or both (Scheidegger et al., 2000; Farquhar et al., 2007). Given that the leaf oxygen isotope (δ^18^O) is unaffected by variability in A, oxygen isotope enrichment in leaf tissue above source water (Δ^18^O) may help in disentangling the independent effects of A and gs on iWUE (Guerrieri et al., 2019).

In this study, we aimed to gain insight into the response of iWUE distribution in coexisting species to aridity gradient, and elucidated the underlying mechanisms. The Loess Plateau (LP) and the Inner Mongolia Plateau (MP) are located mainly in arid and semi-arid regions in China. The preliminary limiting resource for plants is soil N in LP, and water in MP (Ren et al., 2021). Thus, LP and MP provide ideal conditions for an exploration of water use strategies in coexisting species in response to resource-limiting habitats. We hypothesized that (1) increased aridity may lead to a divergence in iWUE distribution, (2) distribution patterns of iWUE along an aridity gradient may be controlled by the most limited resource such as N in LP, and water in MP, and (3) interspecific variation in iWUE is influenced primarily by gs, rather than A, because high VPD and dry soil conditions can lead to severely reduced gs before manifesting in photosynthetic capacity (Utkhao and Yingjajaval, 2015). To test these hypotheses, we established two grassland transects along aridity gradients from east to west in LP and TP. We used bulk leaf δ^13^C and δ^18^O to calculate iWUE and Δ^18^O, respectively. The latter was used to reflect variability in gs among species.

## Materials and methods

### Description of study sites

**Figure 1:**
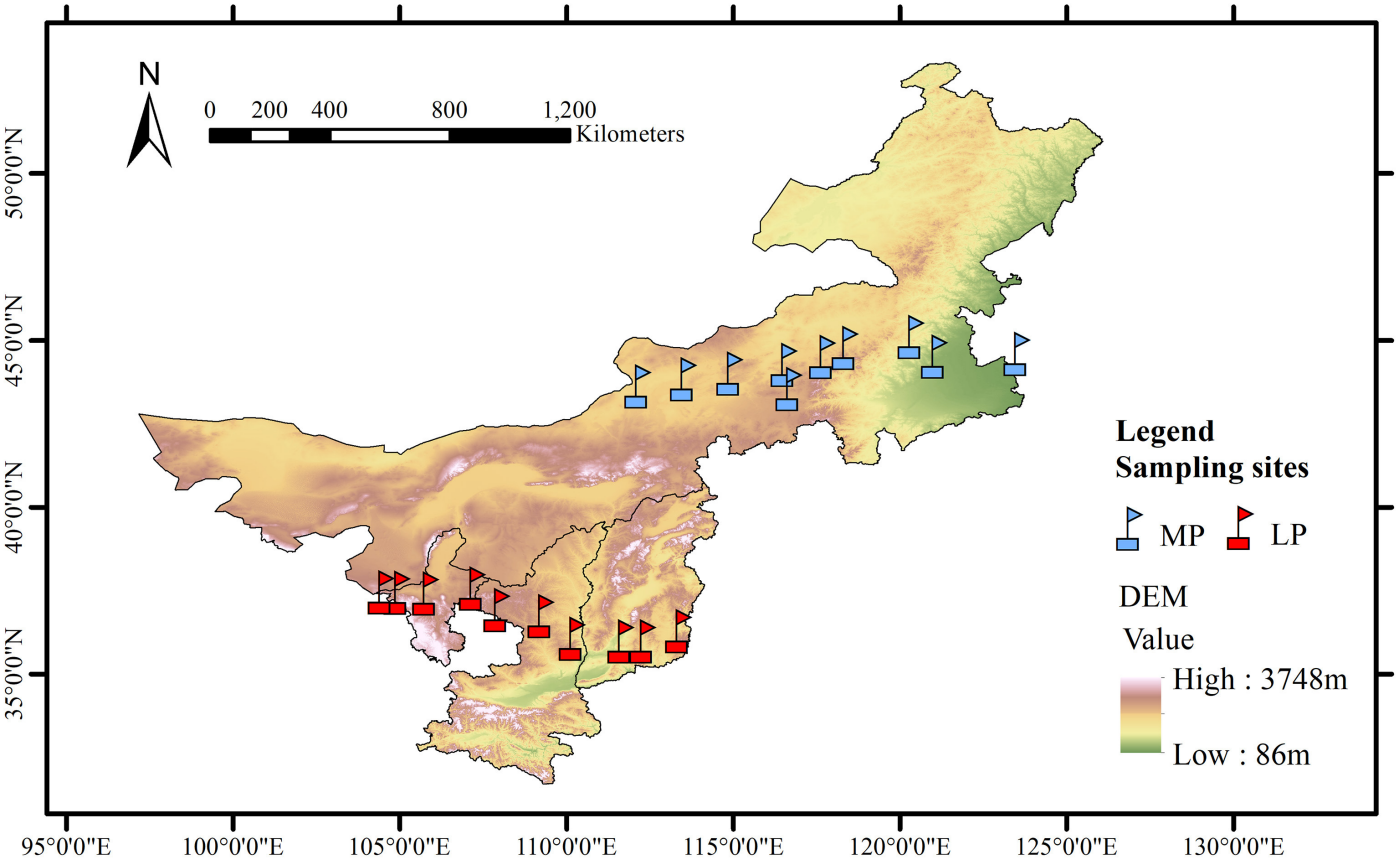
Geographic characteristics of the grassland transects in Loess Plateau (LP) and Inner Mongolia Plateau (MP).

This study was conducted along two grassland transects, one in the Loess Plateau (LP) and one in the Inner Mongolia Plateau (MP) (Figure 1). LP has a typical arid and semi-arid temperate continental monsoon climate, and an extremely fragmented hilly loess landscape due to severe soil and water erosion (Yue et al., 2019). MP experiences arid and semi-arid continental climate.

The LP transect was 600 km long from east (Lucheng District, 113.36°E, 36.29°N) to west (Shapotou District, 104.44°E, 37.46°N), with a wide range in annual total precipitation (189–599 mm) and aridity (0.51–0.63) (Figure 1). Aridity was calculated as 1- mean annual precipitation/ potential evapotranspiration. The MP transect was 1,200 km long from east (Changling County, 123.51°E, 44.59°N) to west (Erenhot, 112.15°E, 43.63°N), with a smaller range in precipitation (183–425 mm) and greater aridity (0.42–0.83). Each transect includes 10 sampling sites at an interval of about 60–150 km from east to west (Lyu et al., 2021). Grassland types include meadow grassland (*n* = 3), typical grassland (*n* = 4), and desert grassland (*n* = 3).

### Sampling and measurements

#### Field survey and sample collection

Field sampling was carried out during the peak growing season (July–August) in 2018. We delineated a 1 km × 1 km sampling area within each sampling site, and tried to collected as many different species as possible (Zhang et al., 2019). Plant species were identified by experienced plant taxonomists. Overall, 574 and 433 plant species were found at LP and MP, respectively, including trees, shrubs, herbs and ferns (Supplementary Table S1).

At least 3 individuals per species per sampling area were selected as replicates. For trees and shrubs, we collected branches from the sunny-side of crowns. For herbs, we collected mature whole plants. Ten, fully expanded and mature leaves (*n* = 50) collected from each species were mixed uniformly into one sample for subsequent analyses. Soil samples were collected from the 0 to 10 cm layer using a soil auger, with eight replications at each site.

#### Leaf functional traits analysis

Leaf apparent morphology was determined using six to 10 fresh leaves per species. Leaf area (LA) was measured with a portable scanner (Cano Scan LIDE 110, Japan). LA values were obtained using an Image J software (Schneider et al., 2012). Leaf dry mass was determined after drying at 60°C. Specific leaf area (SLA) was obtained by dividing LA by leaf dry mass. Then, bulked dried leaf materials were finely ground for subsequent analyses.

Elemental analyzer (Vario Max CN Element Analyser, Elementar, Hanau, Germany) was used to determine bulk-leaf nitrogen (N) content. Leaf N per unit area (N_area_) was calculated from N content and SLA. Elemental analyzer (Model Flash 2000HT, Thermo Fisher Scientific, Bremen, Germany) coupled to an isotope ratio mass spectrometer in continuous-flow mode (Model 253 plus, Thermo Fisher Scientific, Bremen, Germany) was used to analyze carbon (δ^13^C) and oxygen (δ^18^O) isotopes of bulk leaf samples. Isotope ratios are expressed as per mil deviations relative to Vienna Pee Dee Belemnite standard, VPDB for δ^13^C and VSMOW for δ^18^O. Long-term precision for the instrument were <0.1‰ for δ^13^C and <0.2‰ for δ^18^O.

There were 519 and 402 species with bulk leaf δ^13^C < −20‰ (C_3_ species) (Supplementary Table S1), and 55 and 31 species with bulk leaf δ^13^C > −20‰ (C_4_ species), respectively, in LP and MP. In generally, the iWUE of C_4_ species is usually several times higher than that of the C_3_ species (Pinto et al., 2014), and the iWUE of the C_3_ and C_4_ species is not distributed continuously when put together. Consequently, previous studies often investigated C_3_ and C_4_ species separately when analyzing plant water use strategies (Cornwell et al., 2018; Rumman et al., 2018). Given that the number of C_4_ species in the two transects is relatively small, we only selected C_3_ species to elucidated the patterns and underlying mechanisms of iWUE distribution in coexisting species along aridity gradient.

### Calculation of iWUE and Δ^18^O

Time-integrated leaf intrinsic water use efficiency (iWUE, μmol mol^−1^) during the growing season was derived from bulk leaf δ^13^C based on the well-established theory linking CO_2_ partial pressure of leaf intercellular space (Ci) to ambient (Ca) (Ci/Ca) with carbon isotopic carbon discrimination (Δ^13^C) during photosynthesis (Farquhar et al., 1989; Guerrieri et al., 2019). According to Fick's first law of diffusion, iWUE is linearly related to Ci/Ca, and can be expressed as:


(1) $$\begin{array}{l} \text{iWUE}=\text{A}/\text{gs}=\text{Ca}/1.6\times \left(1-\text{Ci/Ca}\right) \end{array}$$

where A is the photosynthetic rate; gs is the stomatal conductance of CO_2_. The Δ^13^C can be calculated as Farquhar et al. (1989):


(2) $$\begin{array}{l} {\text{Δ}}^{13}\text{C}=\left({\;\delta \;}^{\text{13}}{\text{C}}_{\text{a}}-{\;\delta \;}^{\text{13}}{\text{C}}_{\text{L}}\right)/\left(1+{\;\delta \;}^{\text{13}}{\text{C}}_{\text{L}}/1000\right) \end{array}$$

where, δ^13^C_a_ is δ^13^C values of atmospheric CO_2_; δ^13^C_L_ is δ^13^C values of bulk-leaf tissue. According to the “simple” form of isotopic discrimination that does not include effects due to mesophyll conductance and photorespiration (Farquhar et al., 1989; Guerrieri et al., 2019), Ci/Ca can be expressed as:


(3) $$\text{Ci/Ca}=(\Delta^{13}\text{C-a})/(\text{b-a})$$

where a is the isotope fractionation constant during CO_2_ diffusion through stomata (4.4‰); b is the isotope fractionation constant during fixation by Rubisco (27‰). Combining Equation (1) and (3), iWUE can be calculated as follows:


(4) $$\text{iWUE}=\text{Ca}/1.6\times [1\text{-}(\Delta^{13}\text{C-a)/(b-a)}]$$

Oxygen isotope enrichment of leaf tissue above source water (Δ^18^O) was calculated using

Equation (5) (Barbour, 2007):


(5) $$\begin{array}{l} {\text{Δ}}^{18}\text{O}=\left({\;\delta \;}^{\text{18}}{\text{O}}_{\text{L}}-{\;\delta \;}^{\text{18}}{\text{O}}_{\text{S}}\right)/\left(1+{\;\delta \;}^{\text{18}}{\text{O}}_{\text{S}}/1000\right) \end{array}$$

where δ^18^O_L_ and δ^18^O_s_ are oxygen isotope values of bulk leaf and source water, respectively. We assumed that δ^18^O in precipitation reflects that of soil water (i.e., source water), modified by evaporation (Guerrieri et al., 2019).

### Auxiliary dataset

Climate variables for 1970–2000 were extracted from the meteorological database of the WorldClim at 0.1° spatial resolution (https://www.worldclim.org/). Mean annual precipitation and temperature, growing season (April to October) precipitation, temperature and actual water vapor pressure were calculated from monthly values. Aridity was obtained from CGIAR-CSI (https://cgiarcsi.community). Data for soil moisture (SM) (at ~10 cm depth) were obtained from a remote-sensing-based surface soil moisture dataset at 0.1° spatial resolution, and ~10-day temporal resolution (Chen et al., 2021). Vapor pressure deficit (VPD) was derived from actual water vapor pressure and temperature (Grossiord et al., 2020). Soil total N content (TN), and the ratio of soil organic carbon to TN (SOC/TN) were obtained from the “Functional Trait database of terrestrial ecosystems in China (China_Trait).”

### Statistical analysis

Blomberg's K values were used to evaluate the phylogenetic signal of iWUE (Blomberg et al., 2003) using “phytools” and “Picante” package in R software (version 3.5.1, R Development Core version Team, Vienna, Austria), because phylogenetic relationships are an important source of inter-specific differences in leaf functional traits. A significant phylogenetic signal (*P* < 0.05) and large K value indicated that iWUE was constrained by phylogeny (Blomberg et al., 2003). Our results showed that no significant phylogenetic signals of iWUE were observed, and the Blomberg's K values were small (Supplementary Table S2).

Normality of iWUE was tested using the Shapiro–Wilk test (SPSS, Chicago, IL, USA). One-way ANOVA with Duncan's *post hoc* multiple comparisons was used to compare the differences in iWUE among transects and communities, and to partition variances in iWUE within-site from among site (SPSS, Chicago, IL, USA). Multivariate analysis of covariance was used to partition the contribution of sampling site, species and their interaction to variances in iWUE (SPSS, Chicago, IL, USA).

All iWUE values of C_3_ species in each sampling site were used to calculate frequency distribution characteristics, such as mean, variance, standard deviation, coefficients of variation, range, skewness and kurtosis (SPSS, Chicago, IL, USA). Simple linear regressions between frequency distribution characteristics of iWUE and aridity were conducted to test for the first hypothesis.

Pearson correlation analyses were used to test bivariate relationships between SOC/TN, SM, VPD and iWUE (SPSS, Chicago, IL). To test for the hypothesis (2), we conducted variation partitioning (R software, version 3.5.1, R Development Core version Team, Vienna, Austria) to quantify the relative contributions of water (SM and VPD) and SOC/TN to the spatial patterns of iWUE along aridity gradients, combining with the results of Pearson correlation analyses.

Simple linear regressions between iWUE and leaf functional traits (Δ^18^O, SLA and N_area_) were conducted (R2018b, MathWorks, Inc). The result of simple linear regressions between iWUE and Δ^18^O was used to test for hypothesis (3).

A multivariate associations among iWUE, Δ^18^O, SLA, and N_area_ were analyzed with principal component analysis (PCA) (R2018b, MathWorks, Inc) to understand the role of iWUE and Δ^18^O in multidimensional leaf functional traits. Another PCA was performed excluding iWUE and using the remaining three traits (Δ^18^O, SLA, and N_area_). Simple linear regressions between iWUE and axes scores of each species of second PCA were conducted to further explain the drivers of interspecific variation in iWUE along aridity gradient.

## Results

### Distribution of intrinsic water use efficiency

**Figure 2:**
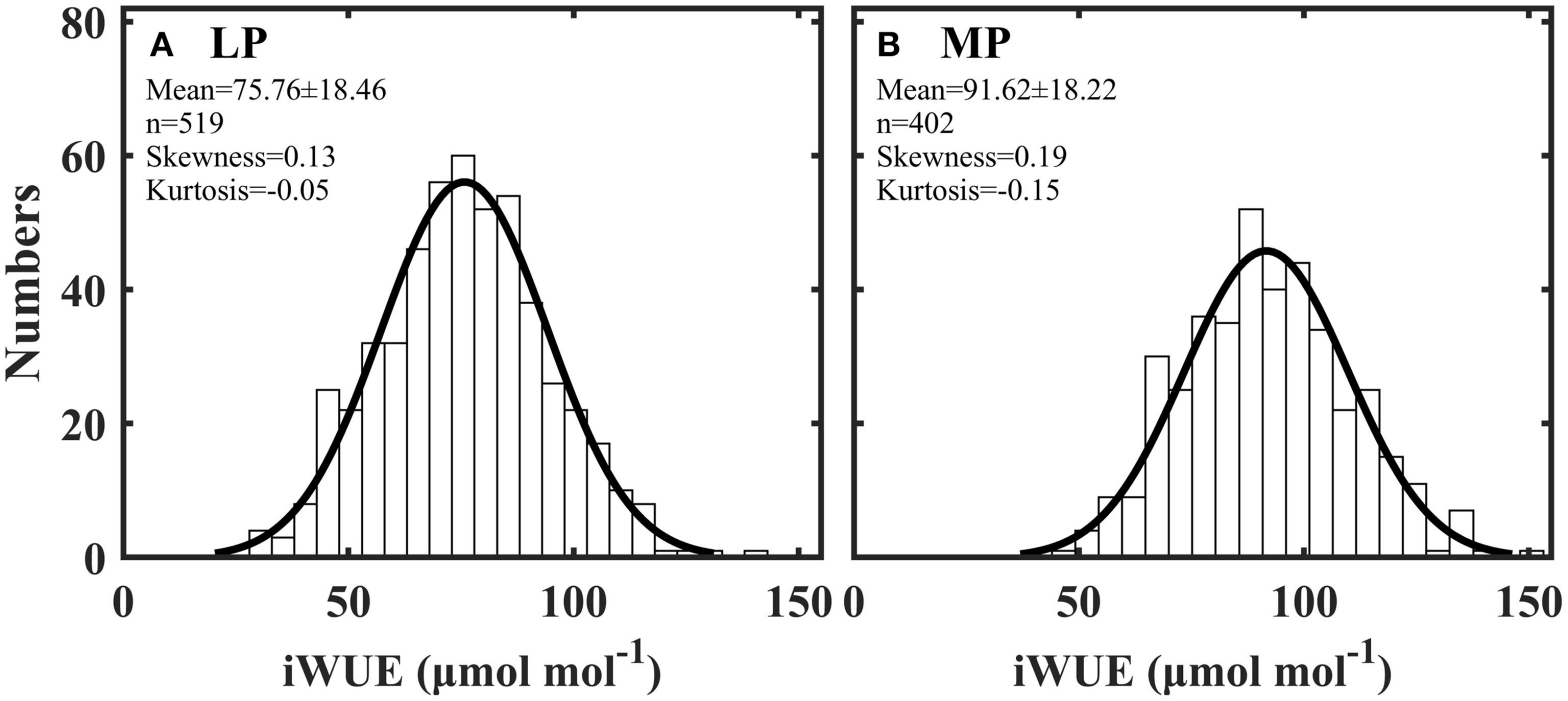
Frequency distribution of intrinsic water use efficiency (iWUE) in Loess Plateau (LP) **(A)** and Inner Mongola Plateau (MP) **(B)**.

Intrinsic water use efficiency (iWUE) was distributed normally across all plant species in Loess (LP) and Inner Mongola (MP) Plateaus (Figures 2A,B). iWUE ranged from 28.17 to 141.97 μmol mol^−1^ in LP and from 47.30 to 152.00 μmol mol^−1^ in MP (Supplementary Table S3). iWUE was significantly lower in LP than in MP (*P* < 0.001).

**Figure 3:**
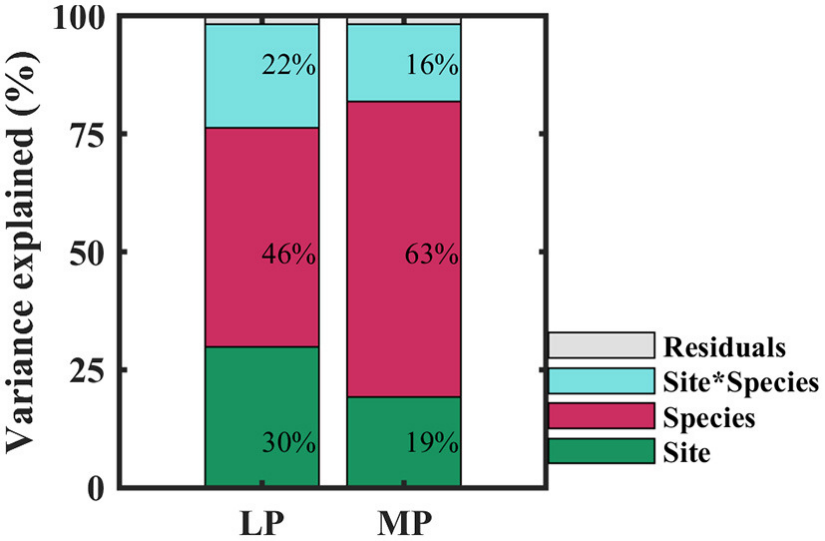
Contributions of species (red), site (green), and their interaction (blue) to variation in intrinsic water use efficiency. LP, Loess Pleateau; MP, Inner Mongolia Plateau.

Within transects, iWUE of co-existing species in each sampling site was distributed normally (*P* > 0.05), except for one sampling site in MP (*P* = 0.03) (Supplementary Table S3). iWUE varied widely within and across sampling sites, and over 60% of variability in iWUE originated from within-site (Supplementary Table S4). When treated as categorical variables, variability in iWUE was primarily controlled by species (46 and 63% in LP and MP, respectively), followed by sampling site (30 and 19% in LP and MP, respectively), and the interaction between species and sites (22 and 16% in LP and MP, respectively) (Figure 3). It indicated that interaction of leaf functional traits and environmental factors drove the patterns of iWUE.

### Drivers of spatial patterns of intrinsic water use efficiency along aridity gradients

**Figure 4:**
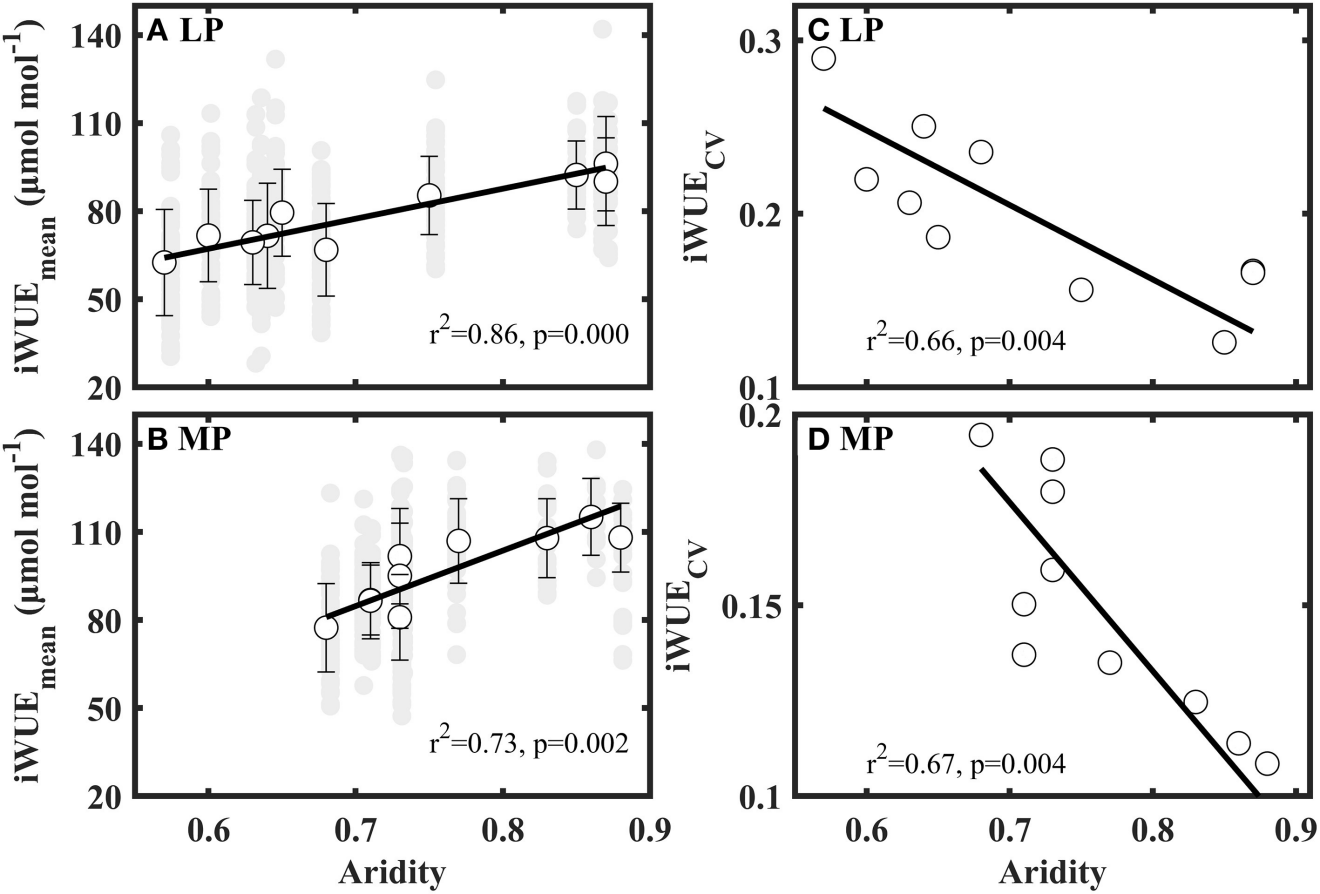
Patterns of mean value (iWUE_mean_) **(A,B)** and coefficient of variation (iWUE_CV_) **(C,D)** of intrinsic water use efficiency (iWUE) along aridity gradients in Loess Plateau (LP) and Inner Mongola Plateau (MP).

Mean values of iWUE (iWUE_Mean_) significantly increased with aridity in LP and MP (Figures 4A,B). Coefficient of variation (CV) of iWUE (iWUE_CV_) decreased with increasing aridity in LP and MP (Figures 4C,D). However, the variance, standard deviation, range, kurtosis, and skewness of iWUE did not show clear patterns along aridity gradients (Supplementary Table S3).

**Figure 5:**
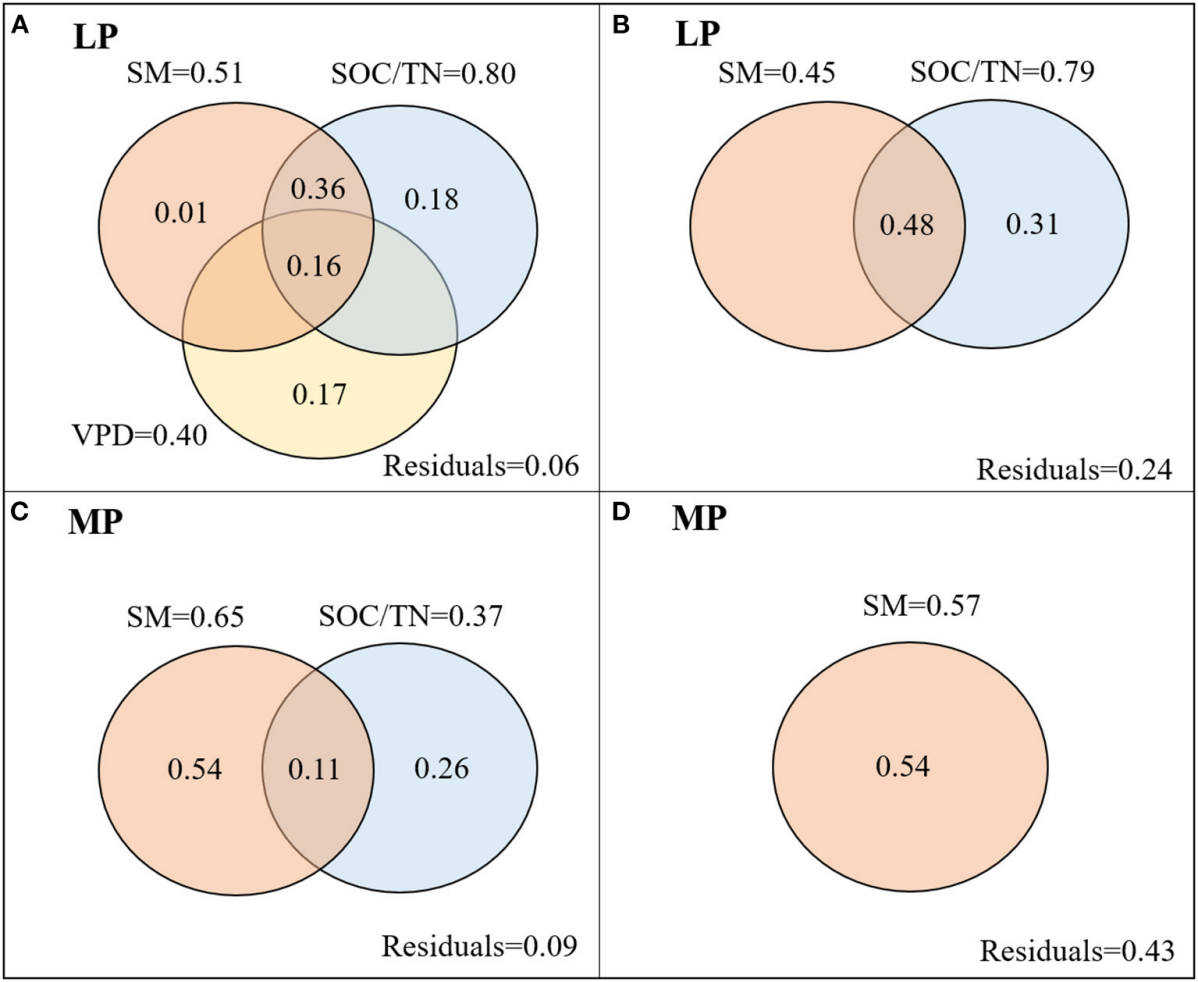
Variation partitioning (R^2^) of environmental factors in mean **(A,C)** and coefficient of variation (CV) **(B,D)** of iWUE of co-occuring species in LP **(A,B)** and MP **(C,D)**. LP, Loess Pleateau; MP, Inner Mongolia Plateau; SM, soil moisture; VPD, vapor pressure deficit, VPD; SOC/TN, the ratio of soil organic carbon content to soil total nitrogen content.

iWUE_Mean_ in LP was negatively related to soil moisture (SM) and to the ratio of soil organic carbon content to total nitrogen content (SOC/TN), and positively related to vapor pressure deficit (VPD) (Table 1). SOC/TN, SM, and VPD explained 94% of variation in iWUE_Mean_, and the strongest predictor was SOC/TN (Figure 5A). iWUE_CV_ was negatively related to SM and SOC/TN (Table 1). SOC/TN and SM explained 76% of variation in iWUE_CV_, and the strongest predictor was SOC/TN (Figure 5B).

**Table 1:** Pearson's correlation coefficients between intrinsic water use efficiency and environmental variables in Loess Plateau (LP) and Inner Mongola Plateau (MP).

		**Loess plateau**		**Inner mongolia plateau**	
		**iWUE**_Mean_	**iWUE**_CV_	**iWUEmean**	**iWUEcv**
Water	SM	−0.746^*^	0.718^*^	−0.828^**^	0.752^*^
	VPD	0.684^*^	−0.476	0.333	−0.496
Nutrient	SOC/TN	−0.905^**^	0.900^**^	−0.663^*^	0.375

*iWUE, intrinsic water use efficiency; CV, coefficient of variation; SM, soil moisture; VPD, vapor pressure deficit, VPD; SOC/TN, the ratio of soil organic carbon content to soil total nitrogen content*.

** and * *represent a significant relationship at p = 0.01 and 0.05 levels, respectively*.

In MP, iWUE_Mean_ was negatively related to SM and SOC/TN (Table 1). SOC/TN and SM jointly explained 91% of variation in iWUE_Mean_, and the strongest predictor was SM (Figure 5C). iWUE_CV_ was positively related to SM (Table 1). SM explained 57% of variation in iWUE_CV_ (Figure 5D).

### Drivers of interspecific variation in intrinsic water use efficiency along aridity gradients

**Figure 6:**
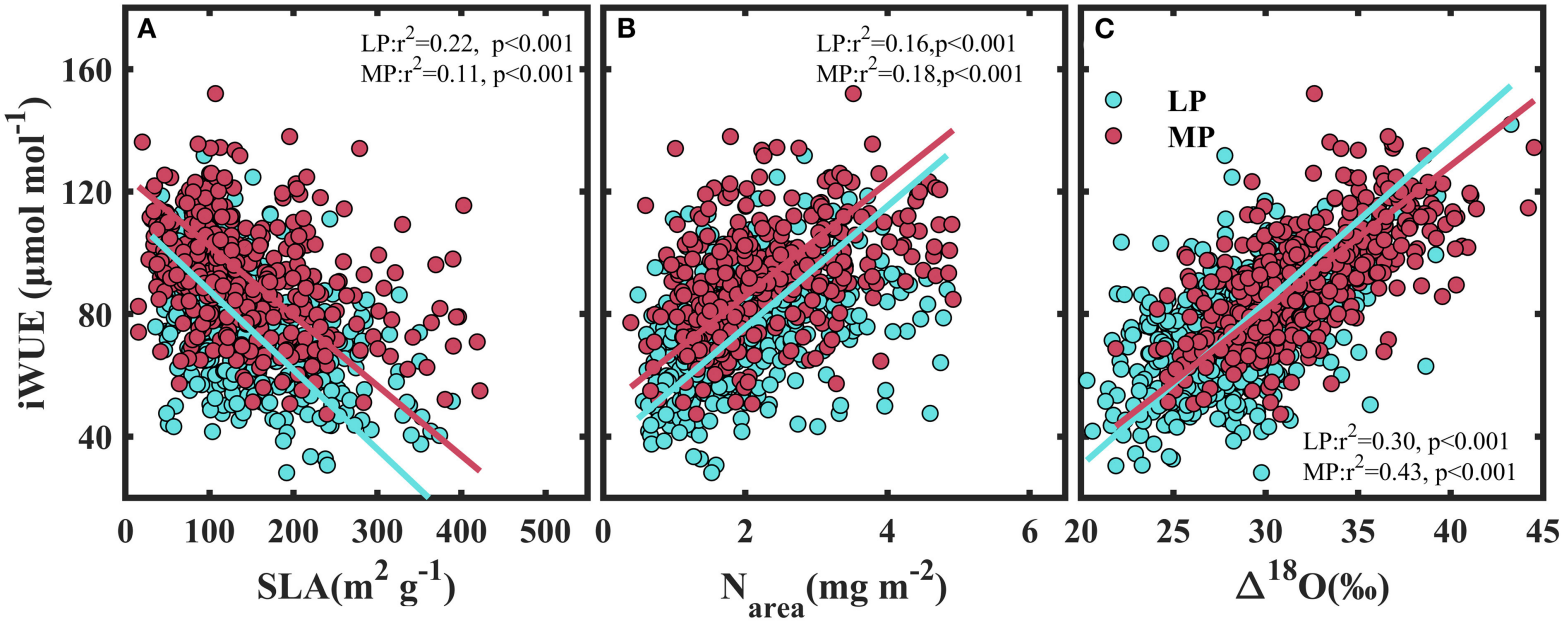
Relationships between intrinsic water use efficiency and specific leaf area (SLA) **(A)**, leaf nitrogen per unit area (N_area_) **(B)**, and the ^18^O enrichment in leaf water above source water (Δ^18^O) **(C)**. LP, Loess Pleateau; MP, Inner Mongolia Plateau.

iWUE decreased with specific leaf area (SLA) (*P* < 0.001) (Figure 6A), and increased with leaf nitrogen per unit area (N_area_) (Figure 6B) and with ^18^O enrichment in leaf water above source water (Δ^18^O) (Figure 6C).

**Figure 7:**
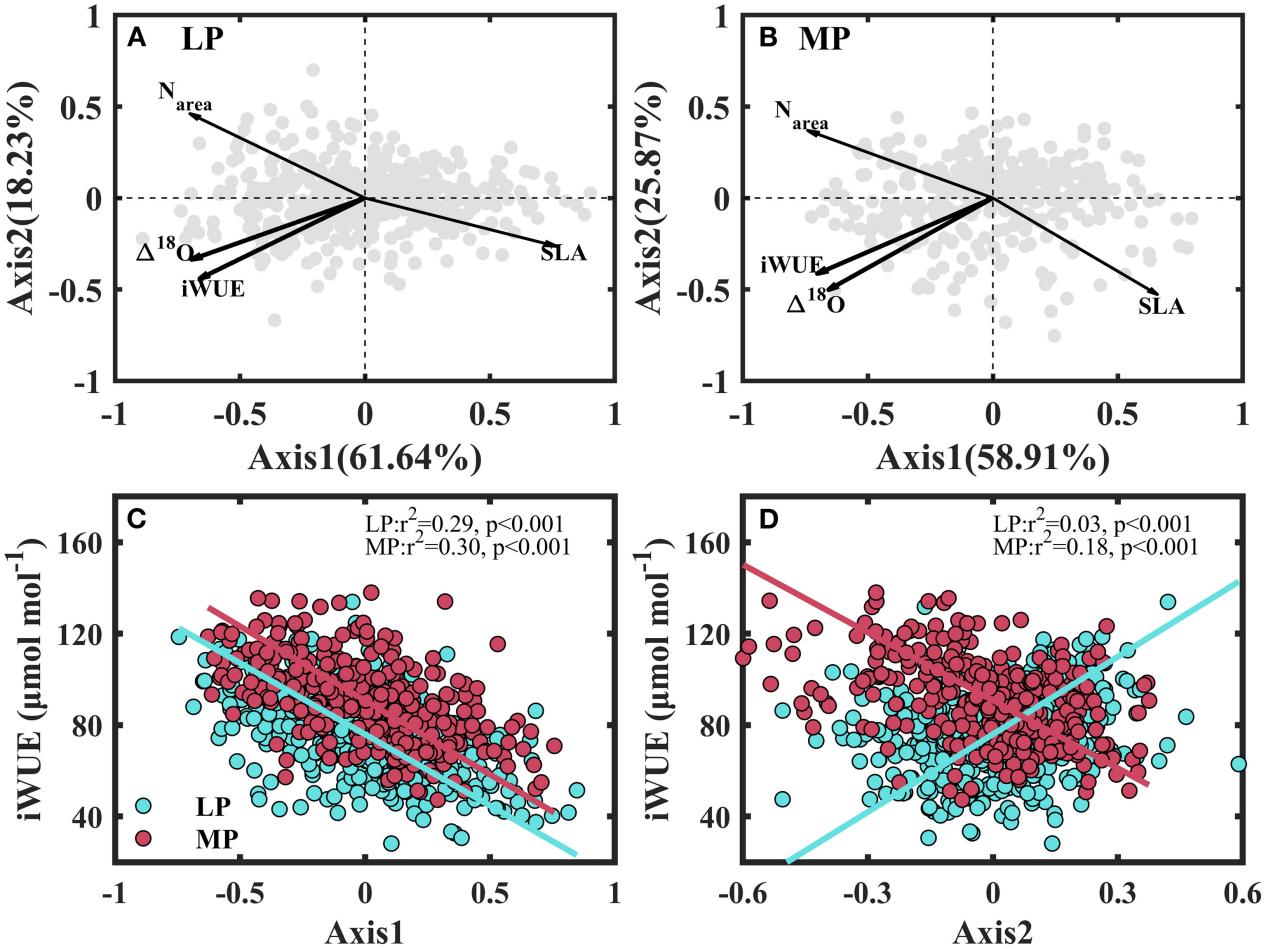
Principal component analysis (PCA) of intrinsic water use efficiency (iWUE) and leaf functional traits **(A,B)** and relationship between the principal component axes of PCA of leaf functional traits and iWUE **(C,D)**. SLA, specific leaf area; N_area_, leaf nitrogen per unit area, and Δ^18^O, the ^18^O enrichment in leaf water above source water. LP, Loess Pleateau; MP, Inner Mongolia Plateau.

Principal component analysis (PCA) was conducted to quantify the effect of plant functional trait combinations on inter-specific variability in iWUE. The dominant leaf functional traits of Axis1 were SLA, Narea, Δ^18^O, and iWUE in LP (Figure 7A and Supplementary Table S5) and MP (Figure 7B and Supplementary Table S5). Axis2 was mainly loaded by N_area_ and iWUE in LP (Figure 7A), and SLA, Δ^18^O, and iWUE in MP (Figure 7B). Variability in iWUE was mainly controlled by Axis1 (*r*^2^ = 0.29) in LP (Figure 7C), and by Axis1 (*r*^2^ = 0.30) and Axis2 (*r*^2^ = 0.18) in MP (Figure 7D).

## Discussion

### Most limited resource led to conservative and convergent water use strategies with an increase in aridity

Species in LP and MP adopt opportunistic water use strategies to cope with water- and nutrient-limiting habitats (Figure 2). Time-integrated iWUE in LP and MP were within the range but distributed at a relatively lower end of global values. For example, two multi-species datasets show that the instantaneous iWUE ranged from 5 to 324.00 μmol mol^−1^ (Flexas et al., 2016; Gago et al., 2016). Furthermore, leaf δ^13^C of species in this study were also distributed at the relatively depleted end of the global δ^13^C dataset (Supplementary Figure S1). The reason may be that an opportunistic water use strategy is advantageous for maximizing nutrient capture (Querejeta et al., 2018; Salazar-Tortosa et al., 2018). Water and nutrient supply in arid and semi-arid habitats are highly heterogeneous in space and time due to the limited and variable rainfall (Chesson et al., 2004). Nutrient uptake from soil is tightly linked to the transpiration-driven mass flow of water (Salazar-Tortosa et al., 2018). Species with low iWUE always exhibit high stomatal conductance (gs) and transpiration rate (Moreno-Gutierrez et al., 2012), allowing rapid nutrient absorption when soil water and nutrients are available.

Within transect, we found that iWUE of co-occuring species in each sampling site exhibited high variability, however, functional convergence was observed along the aridity gradient (Figures 4C,D). This result is contrary to our first hypothesis. Species with diverse and contrasting water use stratergies are known to coexist in dry and nutrient-poor habitats, such as Karst (Wang et al., 2021), Mediterranean (Moreno-Gutierrez et al., 2012), coastal sand dunes (Bermúdez and Retuerto, 2014) and epiphytic (Querejeta et al., 2018) ecosystems. This demonstrates that species differences in eco-physiological function related to the use of resources facilitate species niche segregation (Moreno-Gutierrez et al., 2012; Bermúdez and Retuerto, 2014). There are two mechanisms that might explain the convergent water use strategies along aridity gradient: (1) Inter-specific competition decreased with increasing aridity (Wu et al., 2017). As the number of species (plant species richness) occuring within each community significantly decreases (Supplementary Table S1), available resources for each species may increase (Zhu et al., 2015). (2) The filtering effect of water stress on iWUE of species increased with increasing aridity. The reason is that species with similar physiological parameters can enter a given harsh habitats (Reich et al., 2003; Maracahipes et al., 2018). These results demonsteated that environmental filtering existed within transect, although species exhibited diverse water use strategies in water and nutrient-limited LP and MP.

Futhermore, we also found that co-occuring species within a sampling site shifted their water use strategies from opportunistic (low iWUE and high gs) to conservative (low iWUE and high gs) with an increase in aridity in LP and MP (Figures 4A,B), consistent with previous studies (Zheng and Shangguan, 2007; Liu et al., 2013; Wang et al., 2016; Ale et al., 2018). This reveals that plants lowered their water loss to adopt to the increasing water stress (i.e., low soil moisture and high water vapor pressure deficit) (Supplementary Table S3). However, the slope of the relationship between aridity and mean values of iWUE (iWUE_mean_) in MP (Figure 4B, slope = 102.304) was steeper than that in LP (Figure 4A, slope = 188.901). This indicates that the negative effect of water stress on iWUE is higher in MP than in LP. We attribute this difference to dissimilarity in limited resources between the two transects.

The variability in iWUE_mean_ along an aridity gradient was mainly determined by SOC/TN in LP, and by soil moisture in MP (Figure 5), which supports our second hypothesis. A previous study conducted in this study area demonstrated that plant N availability in LP was lower than that in MP, and increased with aridity in MP, but showed no clear trend in LP (Unpublished data). These results demonstrate that high plant N availability could meet N demand to reduce water cost across the transect in MP (high iWUE and low gs); however, plants in LP promoted N uptake to meet plant N demand at the expense of high water loss (low iWUE and high gs) (Wright et al., 2003). Our findings highlight the importance of balance between acquisitive and conservative strategies along the water and nitrogen gradients: opportunistic water use strategies allows species to established in habits with low soil N habitats due to their high nitorgen acquisition capacity, while conservative water use strategies promote resistance of species to water stress in relatively high soil N habitats (Wright et al., 2003; Maracahipes et al., 2018).

### Interspecific variation in iWUE was primarily regulated by gs

Previous studies demonstrated that Δ^18^O in co-existing species can be used as a reliable indicator of interspecific differences in gs in strongly water-limited ecosystems, and that leaf δ^13^C and δ^18^O can be used to separate the independent effects of photosynthetic rates and gs on iWUE (Moreno-Gutierrez et al., 2012; Querejeta et al., 2018). However, the effects of climatic (vapor pressure deficit [VPD] and temperature) variability and differences in δ^18^O of plant source water on leaf δ^18^O (Δ^18^O) should be considered across large spatial scales (Prieto et al., 2018). In this study, mean values of Δ^18^O in C_3_ species within a sampling site was not affected by VPD or temperature (*P* > 0.05) (Supplementary Table S6). The effect of source water across sites was corrected using δ^18^O of precipitation at each site (Maxwell et al., 2018). However, the water source partitioning in C_3_ species in each sampling site may attenuate the positive correlation between iWUE and Δ^18^O. The slopes of the linear regression between Δ^18^O and iWUE across sites within transect did not differ statistically (Supplementary Figure S2 and Supplementary Table S7). Furthermore, water-source partitioning is not common in grassland communities (Bachmann et al., 2015). Consequently, differences in Δ^18^O in co-occurring species within communities should be determined mainly by the interspecific variation in gs (Wang and Wen, 2022).

A positive relationship was found between Δ^18^O and iWUE both within (Supplementary Figure S2 and Supplementary Table S7) and among sampling sites (Figure 6B), indicating that the variability in iWUE was determined mainly by stomatal regulation (Moreno-Gutierrez et al., 2012; Prieto et al., 2018; Querejeta et al., 2018). This conclusion supported our third hypothesis. Moreno-Gutierrez et al. (2012) found that the large interspecific difference in δ^13^C in a Mediterranean ecosystem was controlled by Δ^18^O rather than photosynthetic rate. However, in this study, a positive relationship between leaf nitrogen per unit area (N_area_) and iWUE indicated that variability in iWUE was also affected by the interspecific variation in photosynthetic rate (Figure 6B), and that species invested more N to compensate for the negative effect of low gs on photosynthetic rates (Flexas et al., 2016). A previous study demonstrated that species with low leaf-specific leaf areas (SLA) had small and thick leaves, which benefit reductions in water loss (Wright et al., 2017). The negative relationship between iWUE and SLA demonstrated that species increased construction costs to prevent water loss (Maxwell et al., 2018).

In this study, the covariation of iWUE, Δ^18^O, SLA and N_area_ in LP and MP (Figure 7) demonstrates that water loss and carbon gain processes were tightly coupled in the study area. It also shows that iWUE and Δ^18^O can be included in the traditional leaf economic spectrum in drylands (Reich, 2014; Prieto et al., 2018). This leaf economic spectrum defined a water use strategy gradient from conservative (high iWUE and low gs) with high N_area_ to profligate (low iWUE and high gs) with high SLA (Prieto et al., 2018). These results revealed that water losses and carbon gain processes in arid and semi-arid regions are tightly coupled (Prieto et al., 2018; Yin et al., 2018; Shi et al., 2021). A second dimension of trait space was observed in LP (N_area_) (Figure 7A) and MP (SLA and Δ^18^O) (Figure 7B), demonstrating that the most limiting resource was N in LP and water in MP (Wang et al., 2021). The limiting-resource spectrum differentiated species along a uniform gradient from resource acquisition to high resource conservative traits (Baltzer and Thomas, 2010; Bermúdez and Retuerto, 2014). Variability in iWUE was mainly controlled by the leaf economic spectrum in LP, and by both—leaf economic spectrum and limiting resource spectrum in MP. This supports the idea that species evolved species-specific strategies to adapt to harsh habitats by partitioning limiting resources (Bermúdez and Retuerto, 2014).

## Conclusions

In this study, we found that species adopted opportunistic water use strategies to adapt to water- and N-limited habitats in arid and semi-arid regions. Variability in iWUE was primarily controlled by species, followed by sampling site, and the interaction between species and sampling site. Within transect, co-occurring species within a sampling site shifted their water use strategies from opportunistic (low iWUE and high gs) to conservative (low iWUE and high gs) with an increase in aridity in LP and MP, and water use strategies of co-occurring species gradually converged. This distribution pattern was driven mainly by the most limited resource, i.e., SOC/TN in LP, and soil moisture in MP. High variability in iWUE mainly determined by stomatal regulation. Furthermore, variability in iWUE was mainly controlled by leaf economic spectrum in LP, and by both—leaf economic spectrum and limiting resource spectrum in MP, indicating that species evolved species-specific strategies to adapt to a limiting habitat by partitioning limiting resources. This study demonstrated that the limiting resource and leaf functional traits jointly determined the distribution patterns of water use strategies of species in LP and MP along an aridity gradient, and emphasized the importance of considering biological processes in dissecting the underlying mechanisms of plant adaptation strategies at large regional scales.

## Data availability statement

The original contributions presented in the study are included in the article/Supplementary material, further inquiries can be directed to the corresponding author/s.

## Author contributions

JW and XW conceived and designed the research. JW conducted stable isotope measurements, data analysis, and wrote the manuscript. XW revised the manuscript. All authors contributed to the article and approved the submitted version.

## Funding

This work was funded by the Strategic Priority Research Program of the Chinese Academy of Sciences (XDA23070202) and National Natural Science Foundation of China (Grant No. 41991234).

## Conflict of interest

The authors declare that the research was conducted in the absence of any commercial or financial relationships that could be construed as a potential conflict of interest.

## Publisher's note

All claims expressed in this article are solely those of the authors and do not necessarily represent those of their affiliated organizations, or those of the publisher, the editors and the reviewers. Any product that may be evaluated in this article, or claim that may be made by its manufacturer, is not guaranteed or endorsed by the publisher.
