# Supplementary material for: Limiting resource and leaf functional traits jointly determine distribution patterns of leaf intrinsic water use efficiency along aridity gradients
Source: Front Plant Sci. 2022 Jul 29;13:909603. doi: 10.3389/fpls.2022.909603 (PMC9372487; doi:10.3389/fpls.2022.909603)
Supplement: Supplementary file 1 [file Data_Sheet_1.docx]

Supplementary Material

# Supplementary Tables

**Supplementary Table S1** Information of C_3_ species in each sampling site in Loess Plateau (LP) and Inner Mongola Plateau (MP). T, Tree; S, Shrub; G, Grass; F, Fern.

| Site | Species number | T | S | G | F | Family number | Mainly Family (≥3, except MP09≥2) |
| --- | --- | --- | --- | --- | --- | --- | --- |
| LP01 | 62 | 7 | 9 | 46 | 0 | 30 | Asteraceae (n=13); Fabaceae (n=9); Asclepiadaceae (n=3); Rosaceae (n=3); |
| LP02 | 58 | 8 | 10 | 40 | 0 | 29 | Asteraceae (n=10); Fabaceae (n=8); Rosaceae (n=5); Chenopodiaceae (n=3);  Lamiaceae (n=3); Linaceae (n=3) |
| LP03 | 60 | 10 | 11 | 38 | 1 | 32 | Asteraceae (n=13); Fabaceae (n=6); Poaceae (n=4); Asclepiadaceae (n=3);  Rosaceae (n=3) |
| LP04 | 68 | 12 | 5 | 51 | 0 | 32 | Asteraceae (n=14); Fabaceae (n=11); Rosaceae (n=4); Chenopodiaceae (n=3) |
| LP05 | 76 | 9 | 13 | 54 | 0 | 28 | Fabaceae (n=13); Rosaceae (n=9); Asteraceae (n=9); Poaceae (n=8);  Chenopodiaceae (n=4); Rhamnaceae (n=3); Liliaceae (n=3) |
| LP06 | 58 | 8 | 6 | 44 | 0 | 25 | Asteraceae (n=12); Poaceae (n=4); Fabaceae (n=4); Rosaceae (n=4);  Asclepiadaceae (n=3); Chenopodiaceae (n=3) |
| LP07 | 53 | 5 | 3 | 45 | 0 | 20 | Asteraceae (n=11); Fabaceae (n=10); Poaceae (n=4); Chenopodiaceae (n=3) ;  Asclepiadaceae (n=3) |
| LP08 | 25 | 2 | 4 | 19 | 0 | 14 | Asteraceae (n=3); Poaceae (n=3); Zygophyllaceae (n=3) |
| LP09 | 36 | 3 | 10 | 23 | 0 | 19 | Asteraceae (n=5); Chenopodiaceae (n=4); Zygophyllaceae (n=3); Liliaceae (n=3) |
| LP10 | 23 | 1 | 7 | 15 | 0 | 11 | Asteraceae (n=5); Zygophyllaceae (n=3); Poaceae (n=3); Chenopodiaceae (n=4) |
|  | 519 | 65 | 78 | 375 | 1 | 240 |  |
| MP01 | 51 | 4 | 8 | 38 | 1 | 27 | Fabaceae (n=7); Asteraceae (n=7); Poaceae (n=4); Rosaceae (n=3);  Chenopodiaceae (n=3) |
| MP02 | 71 | 2 | 9 | 59 | 1 | 26 | Fabaceae (n=12); Poaceae (n=8); Asteraceae (n=7); Liliaceae (n=6);  Rosaceae (n=4); Ranunculaceae (n=4); Lamiaceae (n=3); Chenopodiaceae (n=3) |
| MP03 | 60 | 0 | 5 | 55 | 0 | 21 | Asteraceae (n=12); Rosaceae (n=8); Fabaceae (n=7); Liliaceae (n=5);  Poaceae (n=5); Ranunculaceae (n=3); Iridaceae (n=3); Caryophyllaceae (n=3) |
| MP04 | 42 | 1 | 1 | 40 | 0 | 15 | Asteraceae (n=9); Poaceae (n=7); Liliaceae (n=5); Fabaceae (n=4);  Rosaceae (n=3) |
| MP05 | 40 | 0 | 2 | 38 | 0 | 20 | Liliaceae (n=6); Poaceae (n=6); Chenopodiaceae (n=5); Asteraceae (n=4) |
| MP06 | 54 | 1 | 5 | 48 | 0 | 21 | Fabaceae (n=7); Asteraceae (n=7); Rosaceae (n=6); Liliaceae (n=6);  Poaceae (n=6) |
| MP07 | 34 | 1 | 2 | 31 | 0 | 13 | Poaceae (n=6); Liliaceae (n=5); Rosaceae (n=4); Fabaceae (n=4);  Asteraceae (n=4) |
| MP08 | 24 | 0 | 1 | 23 | 0 | 12 | Poaceae (n=5); Liliaceae (n=4); Fabaceae (n=3); Asteraceae (n=3) |
| MP09 | 10 | 1 | 0 | 9 | 0 | 8 | Liliaceae (n=2); Fabaceae (n=2) |
| MP10 | 17 | 0 | 2 | 15 | 0 | 12 | Asteraceae (n=4) |
|  | 403 | 10 | 35 | 356 | 2 | 175 |  |

**Supplementary Table S2** Parameters of frequency distributions of intrinsic water use efficiency in each sampling site in Loess Plateau (LP) and Inner Mongola Plateau (MP).

| Site | K value | P value | Site | K value | P value |
| --- | --- | --- | --- | --- | --- |
| LP01 | 0.31 | 0.025 | MP01 | 0.19 | 0.134 |
| LP02 | 0.03 | 0.047 | MP02 | 0.04 | 0.454 |
| LP03 | 0.03 | 0.054 | MP03 | 0.24 | 0.078 |
| LP04 | 0.03 | 0.052 | MP04 | 0.12 | 0.526 |
| LP05 | 0.03 | 0.052 | MP05 | 0.13 | 0.065 |
| LP06 | 0.03 | 0.045 | MP06 | 0.13 | 0.090 |
| LP07 | 0.03 | 0.046 | MP07 | 0.13 | 0.146 |
| LP08 | 0.03 | 0.042 | MP08 | 0.14 | 0.409 |
| LP09 | 0.03 | 0.050 | MP09 | 0.38 | 0.448 |
| LP10 | 0.03 | 0.049 | MP10 | 0.23 | 0.274 |
| LP | 0.03 | 0.054 | MP | 0.14 | 0.001 |

**Supplementary Table S3** Parameters of frequency distributions of intrinsic water use efficiency in each site in Loess Plateau (LP) and Inner Mongola Plateau (MP).

|  | n | Mean | Variance | STD | Range | CV | Kurtosis | Skewness | P values |
| --- | --- | --- | --- | --- | --- | --- | --- | --- | --- |
| LP | 519 | 75.76A | 340.91 | 18.46 | 113.79 | 0.24 | 0.13 | -0.05 | 0.43 |
| LP01 | 62 | 62.50e | 327.34 | 18.09 | 75.43 | 0.29 | 0.35 | -0.32 | 0.12 |
| LP02 | 58 | 71.66d | 248.17 | 15.75 | 69.24 | 0.22 | 0.34 | -0.06 | 0.32 |
| LP03 | 60 | 71.58d | 321.22 | 17.92 | 87.95 | 0.25 | 0.05 | -0.33 | 0.85 |
| LP04 | 68 | 69.34de | 204.73 | 14.31 | 84.76 | 0.21 | 0.45 | 1.47 | 0.10 |
| LP05 | 76 | 79.48c | 219.67 | 14.82 | 84.58 | 0.19 | 0.54 | 1.54 | 0.06 |
| LP06 | 58 | 66.81de | 247.72 | 15.74 | 62.13 | 0.24 | 0.17 | -0.91 | 0.24 |
| LP07 | 53 | 85.34bc | 177.55 | 13.32 | 64.40 | 0.16 | 0.47 | 0.39 | 0.29 |
| LP08 | 25 | 92.31a | 135.03 | 11.62 | 43.43 | 0.13 | 0.68 | -0.24 | 0.19 |
| LP09 | 36 | 96.15a | 257.94 | 16.06 | 75.10 | 0.17 | 0.33 | 0.52 | 0.53 |
| LP10 | 23 | 90.06ab | 223.42 | 14.95 | 52.81 | 0.17 | -0.14 | -0.82 | 0.75 |
| Trend | <0.001 | <0.001 | >0.05 | >0.05 | 0.05 | 0.004 | >0.05 | >0.05 |  |
| MP | 402 | 91.62B | 331.86 | 18.22 | 104.69 | 0.20 | 0.19 | -0.15 | 0.24 |
| MP01 | 51 | 77.39e | 226.85 | 15.06 | 72.57 | 0.19 | 0.52 | 0.36 | 0.33 |
| MP02 | 71 | 80.92de | 211.63 | 14.55 | 64.31 | 0.18 | -0.31 | -0.39 | 0.29 |
| MP03 | 60 | 86.78d | 141.44 | 11.89 | 63.50 | 0.14 | 0.18 | 0.41 | 0.86 |
| MP04 | 42 | 86.57d | 169.29 | 13.01 | 45.23 | 0.15 | -0.01 | -1.11 | 0.03 |
| MP05 | 40 | 106.94b | 208.34 | 14.43 | 66.20 | 0.13 | -0.57 | 0.59 | 0.26 |
| MP06 | 54 | 101.74bc | 262.55 | 16.20 | 84.29 | 0.16 | 0.50 | 1.09 | 0.33 |
| MP07 | 34 | 95.05c | 320.01 | 17.89 | 73.55 | 0.19 | 0.41 | -0.08 | 0.60 |
| MP08 | 24 | 107.89ab | 180.92 | 13.45 | 45.62 | 0.12 | 0.28 | -0.93 | 0.32 |
| MP09 | 10 | 115.09a | 171.67 | 13.10 | 43.53 | 0.11 | 0.10 | -0.42 | 0.97 |
| MP10 | 17 | 108.09ab | 292.80 | 11.73 | 56.24 | 0.11 | -0.08 | -1.10 | 0.59 |
| Trend | 0.003 | 0.002 | >0.05 | >0.05 | >0.05 | 0.004 | >0.05 | >0.05 |  |

Note: P values, P values from the Shapiro-Wilk test. Trend, P values for linear regression between aridity and parameters of frequency distributions of intrinsic water use efficiency.

**Supplementary Table S4**Partitioning of the variance of intrinsic water use efficiency within- and among-sampling site.

|  | Within-site (%) | Among-site (%) | P Value |
| --- | --- | --- | --- |
| LP | 70.10 | 29.90 | <0.001 |
| MP | 63.76 | 36.24 | <0.001 |

LP, Loess Plateau; MP, Inner Mongola Plateau.

**Supplementary Table S5** Loading scores of 4 leaf functional traits in the principal components analysis in Loess Plateau and Inner Mongolia Plateau.

|  | Loess Plateau | | Inner Mongolia Plateau | | Loess Plateau | | Inner Mongolia Plateau | |
| --- | --- | --- | --- | --- | --- | --- | --- | --- |
|  | PC1 | PC2 | PC1 | PC2 | PC1 | PC2 | PC1 | PC2 |
| SLA | **0.85** | -0.29 | **0.73** | **-0.59** | 0.89 | 0.14 | 0.86 | -0.38 |
| N_area_ | **-0.78** | **0.51** | **-0.82** | 0.41 | -0.83 | -0.44 | -0.91 | 0.16 |
| △^18^O | **-0.77** | -0.37 | **-0.73** | **-0.56** | -0.75 | **0.65** | -0.59 | **-0.80** |
| iWUE | **-0.73** | -0.49 | **-0.78** | -0.46 |  |  |  |  |
| % of variance | 61.64 | 18.23 | 58.91 | 25.87 | 68.56 | 20.83 | 63.57 | 27.25 |

SLA, specific leaf area; N_area_, leaf nitrogen per unit area, and △^18^O, the ^18^O enrichment in leaf water above source water, iWUE, intrinsic water use efficiency.

**Supplementary Table S6** Pearson’s correlation coefficients for relationships among mean values of ^18^O enrichment in leaf water above source water (△^18^O) in co-existing species within a community and climatic variables

|  | Aridity | Soil moisture  (m^3^ m^-3^) | Vapor pressure deficit  (hPa) | Temperature  (℃) |
| --- | --- | --- | --- | --- |
| Loess Plateau | 0.660* | -0.440 | 0.588 | -0.243 |
| Inner Mongolia Plateau | 0.747* | -0.744* | 0.253 | -0.433 |

**Supplementary Table S7** Coefficients of linear regression between intrinsic water use efficiency and ^18^O enrichment in leaf water above source water (△^18^O) in Losses Plateau (LP) and Inner Mongolia Plateau (MP).

| Sites | Slope | Intercept | r | P value | Sites | Slope | Intercept | r | P value |
| --- | --- | --- | --- | --- | --- | --- | --- | --- | --- |
| LP01 | 5.525 | -79.577 | 0.623 | 0.000 | MP01 | 6.662 | -110.402 | 0.442 | 0.001 |
| LP02 | 7.055 | -137.497 | 0.387 | 0.003 | MP02 | 5.846 | -98.721 | 0.378 | 0.001 |
| LP03 | 5.024 | -72.632 | 0.617 | 0.000 | MP03 | 5.585 | -88.172 | 0.261 | 0.044 |
| LP04 | 5.032 | -75.791 | 0.437 | 0.000 | MP04 | 3.877 | -30.124 | 0.586 | 0.000 |
| LP05 | 4.937 | -58.556 | 0.391 | 0.000 | MP05 | 4.446 | -55.651 | 0.601 | 0.000 |
| LP06 | 5.511 | -81.234 | 0.430 | 0.001 | MP06 | 4.402 | -49.469 | 0.347 | 0.010 |
| LP07 | 4.718 | -55.355 | 0.393 | 0.004 | MP07 | 6.070 | -95.423 | 0.776 | 0.000 |
| LP08 | 3.213 | -5.902 | 0.633 | 0.001 | MP08 | 4.963 | -72.161 | 0.350 | **0.094** |
| LP09 | 3.440 | -7.643 | 0.567 | 0.000 | MP09 | 4.347 | -35.002 | 0.547 | **0.102** |
| LP10 | 4.166 | -32.220 | 0.627 | 0.001 | MP10 | 6.066 | -115.015 | 0.656 | 0.004 |
| LP | 5.334 | -76.213 | 0.546 | 0.000 | MP | 4.703 | -59.598 | 0.654 | 0.000 |

# Supplementary Figures

**Supplementary Figure S1** Frequency distributions of leaf carbon isotope (δ^13^C) in Loess Plateau (LP) (a) and Inner Mongolia Plateau (MP) (b). White bars indicate values from the global dataset.


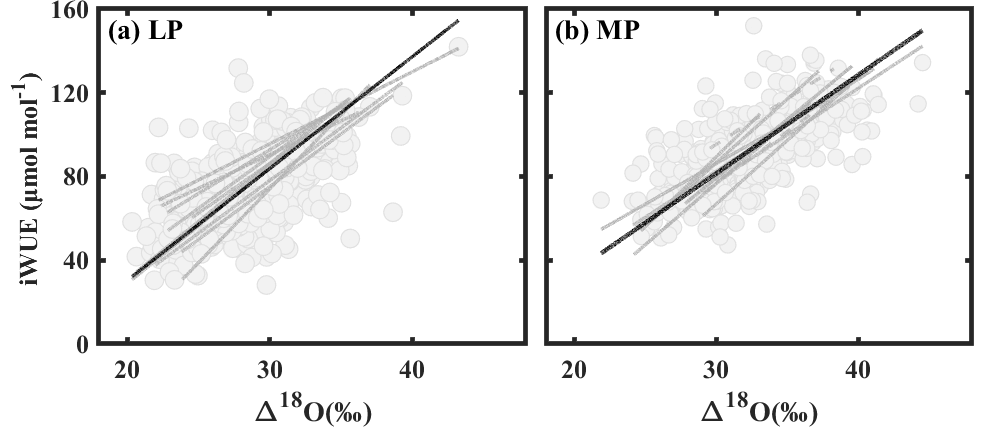


**Supplementary Figure S2** Relationships between leaf ^18^O enrichment in leaf water above source water (△^18^O) and intrinsic water use efficiency (iWUE) in Loess Plateau (LP) (a) and Inner Mongolia Plateau (MP) (b). Black lines show slopes of a linear regression between △^18^O and iWUE for co-occurring species in LP and MP. Grey lines show slopes of a linear regression between △^18^O and iWUE for co-occurring species in a community. Solid lines: P<0.05; Dashed lines: 0.05<P<0.11.
